# Single-centre, single-blind, randomized, active-controlled phase-3 non-inferiority study to investigate the safety and efficacy of the cardioplegic solution Cardioplexol™

**DOI:** 10.3389/fcvm.2025.1587713

**Published:** 2025-05-15

**Authors:** Hendrik T. Tevaearai Stahel, Gabriel Weiss, Peter Landowski, Sandra Folkmann, Marieluise Harrer, Bernard Voet, Martin Grabenwöger

**Affiliations:** ^1^ Cardiovascular Department, Inselspital, Bern University Hospital, University of Bern, Bern, Switzerland; ^2^ Department of Cardiovascular Surgery, Clinic Floridsdorf, Vienna, Austria; ^3^ Karl Landsteiner Institute of Cardiovascular Research, Vienna, Austria; ^4^ Voet Consulting, Berlin, Germany; ^5^ Medical Faculty, Sigmund Freud Private University, Vienna, Austria

**Keywords:** cardiac surgery, cardioplegic solution, cardioplegia, myocardial protection, extracorporeal circulation, randomized controlled trial

## Abstract

**Objectives:**

Effective and reliable cardioplegic cardiac arrest is crucial for maximizing myocardial protection and preserving postoperative contractile function. Aim of this study was to demonstrate, in line with an ongoing European registration procedure, the efficacy and safety of the new Cardioplexol™ solution.

**Methods:**

Single-centre, single-blind, randomized, active-controlled phase-3 non-inferiority trial comparing Cardioplexol™ and Buckberg solutions during cardiac surgery. Patients planed for elective CABG, valve surgery and/or aortic root surgery, were considered eligible after meeting all inclusion and exclusion criteria. Peak troponin-T (TnT) during the first 24 h post-reperfusion was defined as primary endpoint. Intraoperative and ICU-related secondary endpoints were also evaluated, as were safety endpoints.

**Results:**

Out of 248 operated patients, 226 (100 Cardioplexol™, 126 Buckberg) were considered for per-protocol analysis. Peak-TnT was similar in both groups (0.77 vs. 0.78 ng/ml) and non-inferiority of Cardioplexol™ was confirmed. Delay before complete cardiac arrest (11 vs. 71 s, *p* < 0.001) and cross-clamp time (51.2 vs. 60.7 min, *p* < 0.001) were shorter after Cardioplexol™. The defibrillation rate was also significantly reduced (10% vs. 52%, *p* < 0.001). Although not statistically significant, cumulative dose of catecholamines within 24 h postreperfusion (6,202 vs. 7,170 µg/kg, *p* = 0.07), and ICU stay (38.1 vs. 44.0 h, *p* = 0.110) also appeared reduced after Cardioplexol™. Mortality was lower after Cardioplexol™ (1 pt. vs. 5 pts.). Safety parameters were comparable in both groups.

**Conclusion:**

Efficacy and safety of Cardioplexol™ were demonstrated.

**Clinical Trial Registration:**

https://www.clinicaltrialsregister.eu/ctr-search/trial/2011-004198-10/results, Eudra CT-No: 2011-004198-10.

## Introduction

Most cardiac surgery procedures require efficient and reliable cardiac arrest to ensure heart inactivity, while guaranteeing maximal myocardial protection to preserve post-operative contractile function. Several cardioplegic solutions were developed over the last 50 years, such as Buckberg, St-Thomas or Bretschneider solutions (1), most of them being still considered as standard (2–4). Surprisingly, only a handful of cardioplegic solutions are approved by regulatory authorities in some countries. In addition, several centers modify standard solutions or manufacture a customized cardioplegia (5–7). Therefore, even if numerous studies have reported on clinical results obtained with these various solutions, only very few reach a sufficient level of evidence (8).

Cardioplexol™ is a ready-to-use new cardioplegic solution combining four well characterized chemical ingredients at pharmacologically compatible doses. With its low volume (100 ml), the solution was originally conceived to match with the concept of Minimal invasive Extra Corporeal Circulation (MiECC) which consists of a closed circuit operating at reduced and constant volume (9). With experience, it appeared valuable in regular procedures as well. At the time of study preparation, Cardioplexol™ had been used in Bern, Switzerland, in ∼5,000 patients, demonstrating several advantages and no noticeable sideeffect (9–11).

The aim of this pivotal study was therefore to demonstrate, in the context of a European registration procedure, the safety and efficacy of Cardioplexol™.

## Patients and methods

### Study design

Single-centre, single-blind, randomized, active-controlled phase-3 noninferiority trial investigating the safety and efficacy of Cardioplexol™ during cardiac surgeries performed with a heart-lung machine. The study is part of a national registration procedure in Switzerland and a decentralized procedure (DCP) in Europe (RMS: Austria), and was conducted at the Hospital Hietzing, Vienna, between May-2012 and July-2015.

Study protocol was approved by the Ethics Committee of the City of Vienna (EK-11-191-1011, February 17, 2012) and the Austrian regulatory agencies BASG and AGES. Each patient provided written informed consent preoperatively.

### Investigational drug

Cardioplexol™ is a two-component system: vial-A (95 ml) and syringe-B (5 ml). The final 100 ml solution, obtained by injecting the syringe content into the vial, contains potassium-chloride (10.0 mmol), magnesium-sulfate-heptahydrate (16.2 mmol), xylitol (29.6 mmol) and procaine-hydrochloride (1.1 mmol), is hypertonic (850 mosmol) and slightly acidic (pH ∼ 6.0).

Boxes of Cardioplexol™ are kept refrigerated (2–8°C) before use. The final solution is prepared short before use and kept in 2 sterile 50ml-syringes on ice on the instruments' table, ready for injection.

The initial dose of Cardioplexol™ is administered immediately after aortic crossclamping. The surgeon connects the first 50 ml syringe to the cardioplegia cannula, gently aspirates a few ml of blood to check the connection and complete venting of the cannula, then rapidly injects the entire content (5–7 s) into the aortic root. The procedure is immediately repeated with the second syringe. In situations where aortic cross-clamping is likely to last longer than 60 min, a second dose of Cardioplexol™ (50–100 ml) must be administered between 45 and maximal 60 min of cross-clamping. Similarly, if aortic clamping is likely to last longer than 90 respectively 120 min, a third respectively fourth dose (50–100 ml) must be administered after 75 (maximal 90) respectively 105 (maximal 120) minutes of clamping.

### Comparative medicine

After consulting Austrian and German drug registration authorities, it was decided to compare Cardioplexol™ with Buckberg blood cardioplegia (12), considered a benchmark (13, 14). Buckberg was supplied by Dr. Franz Köhler Chemie GmbH (Alsbach-Hähnlein, Germany).

### Study populations and randomization

Every patient, aged 18–80, with indication for elective CABG and/or valve replacement/repair and/or aortic root surgery, planned to be performed under with ECC, was considered eligible for enrollment. Exclusion criteria: preoperative LVEF < 30%, IABP, catecholamine support, myocardial infarction within 7 days, previous cardiac surgery including pace-maker or ICD, active myocarditis and/or endocarditis, aortic valve insufficiency (severity grade >1), history of atrial fibrillation or neurologic event, known carotid artery disease, HIT, dialysis or pre-operative creatinine >2.0 mg/dl, anti-vitamin K treatment or known hematologic disorder, patient is pregnant or lactating, intravenous drug users, alcohol abusers, prisoners, patients institutionalized or unable to give informed consent.

Following informed consent, at least 24 h prior to surgery, eligible patients were randomly assigned to Cardioplexol™ or Buckberg cardioplegia in a 1:1 ratio. Allocation sequence was correctly concealed using web-based central unrestricted randomization (WebSpirit, 2 mt Inc. Ulm, Germany), stratified according to surgical indication.

### Surgical procedure

After usual cardiac exposure and cannulation, a cardioplegia cannula was inserted into the aorta, connected to a three-way valve and de-aired. CPB was started and increased to 100% flow. The ascending aorta was then clamped after checking that cardiac chambers are adequately unloaded. Cardioplegic solution was administered, and the intervention proceeded as usual.

### Study endpoints

Post-operative troponin-T (TnT) is considered a suitable parameter that adequately reflects myocardial preservation and effectiveness of cardioplegia (15–17). Therefore, peak value of TnT during the first 24 h following myocardial reperfusion was set as primary endpoint. Values were assessed at 6, 12 and 24 h post reperfusion. Secondary endpoints are listed in Table 1. Safety endpoints included serious and non-serious adverse events and laboratory values.

**Table 1:** Description of primary and secondary endpoints.

Primary endpoint	
	Maximal value of troponin T (TnT) value during the first 24 h following myocardial reperfusion
Major secondary endpoint	
1	Maximal value of creatinine kinase isoenzyme muscle-brain (CK-MB) during the first 24 h following myocardial reperfusion
Intra-operative related secondary endpoints	
2	Time between the aortic cross-clamping and the complete cardiac arrest
3	Percentage of patients requiring catecholamines during aortic cross-clamping
4	Cumulative dose of catecholamines during aortic cross-clamping
5	Defibrillation rate after aorta unclamping and coronary reperfusion
ICU related secondary endpoints	
6	Cumulative dose of catecholamines during the first 24 h following coronary reperfusion or until ICU discharge (if discharge occurs before 24 h), starting the calculation at arrival to ICU
7	Percentage of patients requiring the installation of an IABP during the first 24 h following coronary reperfusion or until ICU discharge (if discharge occurs before 24 h).
8	Duration of intubation
9	Duration of ICU stay
10	Mortality during the first 24 h following coronary reperfusion or until ICU discharge (if discharge occurs before 24 h)
11	Maximal ST elevation during the first 24 h following coronary reperfusion or until ICU discharge (if discharge occurs before 24 h)
Follow-up related secondary endpoints	
12	Duration of hospitalization.
Safety endpoints	
1	Serious and non-serious adverse events
2	Laboratory parameters

### Sample size calculation and statistical analysis

Based on a similar population analysis, it was estimated that 260 patients would be required to achieve 240 completed patients. Non-inferiority margin was set at 20% above the value reported for Buckberg solution. The sample size of 120 in each group was sufficient with a 1:1 allocation, a power of 80% and a twosided 95% CI. Cardioplexol™ was considered as non-inferior to Buckberg if the upper boundary of the two-sided 95% CI for the ratio of TnT values was below this margin.

Descriptive statistics are expressed as mean, standard deviation, median and ranges for continuous variables, and frequencies and percentages for categorical variables. All statistical tests were two-sided, and a *p*-value of <0.05 was considered significant. Log-transformed TnT and CK-MB values were used for statistical analyses. Both groups were assessed using t-test for continuous variables and Fisher's exact test for dichotomous variables.

### Analysis sets

- Screening population: All patients who gave informed consent.
- Full-Analysis-Set (FAS): All eligible patients who were operated and received the study treatment.
- Safety population: All patients of the FAS-population.
- Per-Protocol-Set (PPS): All subjects of the FAS-population with non-missing max. TnT values and without protocol deviations.
- Modified-Per-Protocol-Set (mod-PPS): PPS-population with protocol deviations considered clinically irrelevant.

## Results

### Patient disposition, demographic and pre-operative characteristics

**Figure 1:**
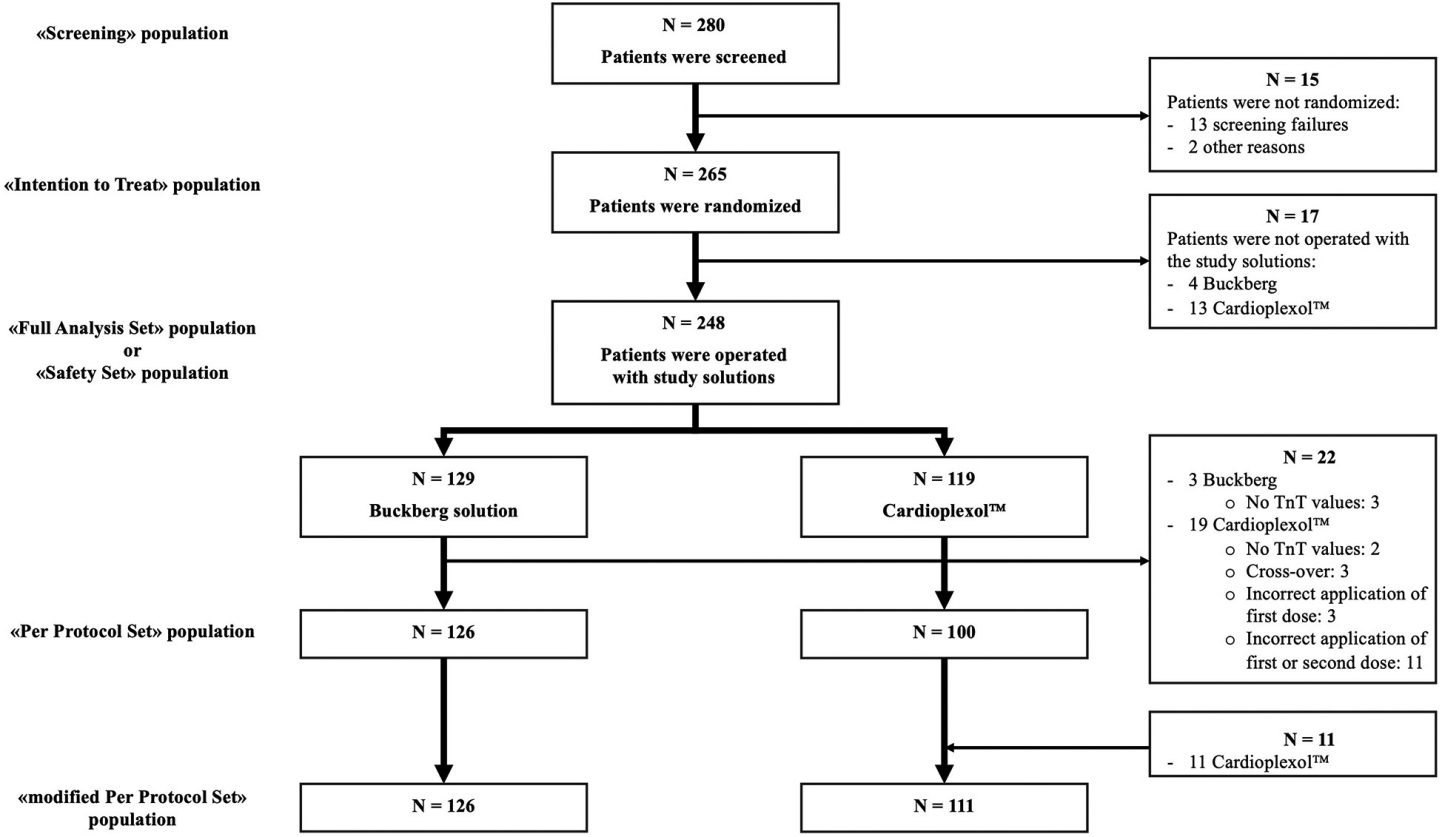
Patients disposition.

Overall, 280 patients were screened. Fifteen were excluded before randomization (Figure 1): in-/exclusion criteria (*n* = 13), patient's refusal (*n* = 1), surgeon's decision to exclude (*n* = 1).

Of 265 randomized patients, 17 were not operated (4 Buckberg, 13 Cardioplexol™; Supplementary Appendix 1). Therefore, 248 patients (FAS), were operated and received either Buckberg (*n* = 129) or Cardioplexol™ (*n* = 119). Baseline profiles are summarized in Table 2.

**Table 2:** Patients’ demographic and pre-operative characteristics (FAS 604 population).

	Cardioplexol™ (*N* = 119)	Buckberg (*N* = 129)
Demographics		
Age (years)	66.2 ± 9.26	65.7 ± 8.94
Gender (male)	85 (71.4%)	99 (76.7%)
Height (cm)	171.0 ± 9.39	172.1 ± 7.89
Weight (kg)	82.0 ± 16.77	84.8 ± 14.80
BMI (kg/m^2^)	27.9 ± 4.51	28.6 ± 4.74
Patient risk factors		
Smoker		
Current	17 (14.3%)	24 (18.6%)
Former	46 (38.7%)	52 (40.3%)
Never	53 (44.5%)	52 (40.3%)
Unknown	3 (2.5%)	1 (0.8%)
Diabetes (yes)	35 (29.4%)	46 (35.7%)
Dyslipidemia (yes)	107 (89.9%)	116 (89.9%)
Systemic hypertension (yes)	108 (90.8%)	118 (91.5%)
Coronary artery disease (yes)	101 (84.9%)	107 (82.9%)
Cerebrovascular disease (yes)	20 (16.8%)	22 (17.1%)
Neurologic dysfunction (yes)	4 (3.4%)	2 (1.6%)
Allergy (yes)	37 (31.1%)	42 (32.6%)
Logistic Euroscore	3.36 ± 2.779	3.21 ± 2.476
NYHA class		
I	1 (0.8%)	2 (1.6%)
II	17 (14.3%)	12 (9.3%)
III	15 (12.6%)	21 (16.3%)
IV	1 (0.8%)	0
Unknown	85 (71.4%)	94 (72.9 0%)
Patient risk factors		
Normal coronaries (yes)	21 (17.6%)	21 (16.3%)
LM stenosis >50% (yes)	25 (21.0%)	10 (7.8%)
Number of diseased vessels		
1	8 (6.7%)	10 (7.8%)
2	1 (16.0%)	18 (14.0%)
3	71 (59.7%)	78 (60.5%)
Unknown	21 (17.6%)	23 (17.8%)
Left ventricular contractile function		
Good (>50% EF)	104 (87.4%)	110 (85.3%)
Moderate (30–50% EF)	14 (11.8%)	18 (14.0%)
Poor (<30%)	0	1 (0.8%)
Unknown	1 (0.8%)	0
Current angina (yes)	57 (47.9%)	59 (45.7%)
History of myocardial infarction >7 days prior to surgery (yes)	33 (27.7%)	41 (31.8%)
Arrhythmia (other than atrial fibrillation) (yes)	3 (2.5%)	5 (3.9%)
Cardiac base rhythm is a sinus rhythm (yes)	113 (95.0%)	122 (94.6%)

Of these FAS patients, another 22 (3 Buckberg, 19 Cardioplexol™) had to be excluded (5 patients without post-operative TnT value, 3 who received Buckberg's solution in addition to Cardioplexol™, and 14 for whom Cardioplexol™ was not administered according to protocol, Supplementary Appendix 2), resulting in a PPS-population of 226 patients (126 Buckberg, 100 Cardioplexol™).

### Modified-PPS-population

The large number of Cardioplexol™ patients excluded from the PPS-population was mainly due to a strict interpretation of the administration protocol, which states that second and third doses of Cardioplexol™ must be administered within a 60- and 90-minute time limit respectively. In nine cases, clamping time exceeded 60 min, without a second dose being administered. Careful analysis (Supplementary Appendix 3) shows, however, that this limit was exceeded by less than 3 min in 5 patients, and less than 10 min in 2 others. For the first 5 patients, corresponding post-operative max.-TnT values were all below 1.0 ng/ml. For longer procedures, TnT values increased progressively. In two other cases, clamping time extended beyond 90 min without a third dose. In a real-life setting, surgeons might regard these times as indicative rather than strict, and minimal deviations would be considered uncritical. Consequently, the 11 patients for whom timing of second or third dose administration was not strictly adhered to were re-entered in a newly defined “mod-PPS” population, thus minimizing differences in patient numbers between Cardioplexol™ (*n* = 111) and Buckberg (*n* = 126) groups. Eventually, only eight Cardioplexol™ patients were excluded from mod-PPS: major administration problem (*n* = 3), cross-over to Buckberg (*n* = 3), no postoperative TnT values (*n* = 2).

### Surgical characteristics

Types of procedures and surgical caracteristics were comparable between the groups (Table 3). However, ECC- and cross-clamp times were shortened by 67 min among Cardioplexol™ patients and 63% required a single dose. Conversely, Buckberg patients required up to 7 doses. Duration (18.6 ± 19.5 vs. 250.6 ± 78.5 s for the first dose, and 9.9 ± 8.6 vs. 123.6 ± 30.2 s for the second dose) and volume of injections (102.8 ± 13.6 vs. 271.4 ± 73.7 ml for the first dose, and 63.6 ± 22.5 vs. 123.3 ± 42.4 ml for the second dose) were also markedly reduced after Cardioplexol™.

**Table 3:** Patients’ operative characteristics (FAS population).

	Cardioplexol™ (*N* = 119)	Buckberg (*N* = 129)
CABG	95 (79.8%)	104 (80.6%)
1×CABG	10 (8.4%)	11 (8.5%)
2×CABG	17 (14.3%)	22 (17.1%)
3×CABG	55 (46.2%)	56 (43.4%)
4×CABG	13 (10.9%)	15 (11.6%)
Aortic valve replacement	34 (28.6%)	34 (26.4%)
Mitral valve repair	2 (1.7%)	3 (2.3%)
Aortic root repair	3 (2.5%)	3 (2.3%)
ECC time (min)	89.9 ± 22.70	96.1 ± 26.79
Cross-clamp time (min)	54.2 ± 15.71	60.9 ± 20.54
£ 60 min	80 (67.2%)	66 (51.2%)
>60 min	37 (31.1%)	60 (46.5%)
Unknown	2 (1.7%)	3 (2.3%)
Doses		
1	75 (63.0%)	4 (3.1%)
2	40 (33.6%)	11 (8.5%)
3	4 (3.4%)	27 (20.9%)
4	0	53 (41.1%)
5	0	22 (17.1%)
6	0	7 (5.4%)
7	0	5 (3.9%)
Duration of initial dose injection (seconds)	18.6 ± 19.49	250.0 ± 78.53
Volume of initial dose (ml)	102.8 ± 13.59	271.4 ± 73.75
Duration of second dose injection (s)	9.9 ± 8.61	123.3 ± 30.15
Volume of second dose (ml)	63.6 ± 22.53	123.3 ± 42.37
Total Volume of cardioplegia (ml)	128.4 ± 40.90	645.0 ± 221.73

### Surgeon's influence

Each surgeon included comparable numbers of patients in both groups, except for two who included only 3, respectively 1 patient. Five surgeons included at least 20 patients, whereas 7 including less than 20 (Table 4). Excluded cases and administration errors were equally distributed between participating surgeons.

**Table 4:** Summary of numbers of cases included and excluded by 613 participating surgeons (FAS population).

	Cardioplegic solution	Total included in FAS	Total not included in PPS	Reasons
Surgeon-1	Buckberg	5	0	–
	Cardioplexol™	7	2	Incorrect timing (1) Incorrect volume (1)
Surgeon-2	Buckberg	4	0	–
	Cardioplexol™	4	0	–
Surgeon-3	Buckberg	49	1	Missing TnT
	Cardioplexol™	35	1	Incorrect timing
Surgeon-4	Buckberg	10	1	Missing TnT
	Cardioplexol™	13	4	Cross-over (2)^a^
				Incorrect timing (2)
Surgeon-5	Buckberg	3	0	–
	Cardioplexol™	0	0	–
Surgeon-6	Buckberg	14	0	–
	Cardioplexol™	11	3	Incorrect timing (2)
				Incorrect volume (1)
Surgeon-7	Buckberg	1	0	–
	Cardioplexol™	0	0	
Surgeon-8	Buckberg	7	1	Missing TnT
	Cardioplexol™	6	1	Missing TnT
Surgeon-9	Buckberg	3	0	–
	Cardioplexol™	6	0	–
Surgeon-10	Buckberg	14	0	–
	Cardioplexol™	17	4	Cross-over (1)^a^
				Missing TnT (1)
				Incorrect timing (2)
Surgeon-11	Buckberg	5	0	–
	Cardioplexol™	5	1	Incorrect duration
Surgeon-12	Buckberg	14	0	–
	Cardioplexol™	15	3	Incorrect timing (3)

^a^ Patient received Buckberg in addition to Cardioplexol™.

Of 10 surgeons who operated with both cardioplegia, 5 had lower TnT results with Cardioplexol™, while the other had lower TnT results with Buckberg (Table 5).

**Table 5:** Comparison of max. TnT values (ng/ml) obtained by the 12 participating surgeons (FAS population).

Surgeon	Buckberg (*n* = 129)			Cardioplexol™ (*n* = 119)		
	*n*	mean ± SD	median (IQR)	*n*	mean ± SD	median (IQR)
Surgeon-1	5	0.55 ± 0.14	0.64 (0.46–0.65)	7	2.49 ± 3.56	0.75 (0.66–4.18)
Surgeon-2	4	1.70 ± 0.71	1.57 (1.12–2.27)	4	0.83 ± 0.20	0.79 (0.68–0.99)
Surgeon-3	48	0.87 ± 0.51	0.75 (0.54–1.04)	35	1.08 ± 0.68	0.94 (0.59–1.44)
Surgeon-4	9	0.76 ± 0.34	0.85 (0.42–1.10)	13	1.44 ± 2.21	0.70 (0.59–1.23
					0.32	
Surgeon-5	3	0.74 ± 0.24	0.78 (0.48–0.95)	0	–	–
Surgeon-6	14	1.16 ± 0.70	1.05 (0.56–1.56)	11	1.09 ± 0.83	0.97 (0.57–1.08)
Surgeon-7	1	0.13	0.13	0	–	–
Surgeon-8	6	0.80 ± 0.65	0.47 (0.39–1.19)	5	0.89 ± 0.36	0.74 (0.71–0.96)
Surgeon-9	3	0.84 ± 0.73	0.48 (0.35–1.68)	6	0.86 ± 0.82	0.55 (0.40–0.84)
Surgeon-10	14	0.83 ± 0.54	0.72 (0.42–1.03)	16	0.80 ± 0.50	0.71 (0.41–1.07)
Surgeon-11	5	0.93 ± 0.64	1.19 (0.28–1.23)	5	1.60 ± 1.69	1.04 (0.74–1.14)
Surgeon-12	14	1.56 ± 1.67	1.02 (0.58–1.52)	15	0.80 ± 0.44	0.80 (0.42–1.17)

For 3 patients in the Buckberg group and 2 in the Cardioplexol™ group, no post-operative TnT value was collected.

### Primary endpoint

Results of max.-TnT values are presented in Table 6.1 for FAS-, PPS- and mod-PPS-populations. Values after 6 h of myocardial reperfusion are presented in Table 6.2.

**Table 6.1:** Non-inferiority analysis of max.-TnT results (ng/ml).

	Cardioplexol™ (*n* = 119)		Buckberg (*n* = 129)		Comparison
	*n*	TnT Mean (95% CI)	*n*	TnT Mean (95% CI)	Ratio (95% CI)
FAS	117^a^	0.84 (0.75–0.95)	126^a^	0.78 (0.70–0.87)	1.08 (0.91–1.28)
PPS	100	0.77 (0.68–0.87)	126	0.78 (0.70–0.87)	0.99 (0.84–1.16)
Mod-PPS	111	0.79 (0.71–0.89)	126	0.78 (0.70–0.87)	1.02 (0.87–1.19)

^a^ 5 patients (2 Cardioplexol™, 3 Buckberg) had no troponin values post-surgery.

**Table 6.2:** Non-inferiority analysis of TnT results at 6 h post-reperfusion (ng/ml).

	Cardioplexol™ (*n* = 119)		Buckberg (*n* = 129)		Comparison
	*n*	TnT Mean (95% CI)	*N*	TnT Mean (95% CI)	Ratio (95% CI)
FAS	116^a^	0.71 (0.62–0.81)	120^a^	0.78 (0.68–0.88)	0.91 (0.75–1.11)
PPS	99	0.64 (0.56–0.74)	120	0.78 (0.68–0.88)	0.83 (0.68–1.00)
Mod-PPS	110	0.67 (0.59–0.77)	120	0.78 (0.68–0.88)	0.87 (0.72–1.05)

^a^ 12 patients (3 Cardioplexol™, 9 Buckberg) had no troponin values at 6 h post-surgery.

Subgroups were analyzed separately for possible differences by age category (Table 7), gender (Table 8) or type of surgery (Table 9).

**Table 7.1:** Comparison of max. TnT values (ng/ml) in various age groups: FAS population.

Age	Allocation	*n*	Mean ± SD	Median (IQR)
<65	Buckberg	51	0.791 ± 0.414	0.680 (0.480–1.070)
	Cardioplexol™	39	1.343 ± 1.726	0.900 (0.590–1.310)
	Total	90	1.030 ± 1.201	0.725 (0.500–1.100)
≥65 and <70	Buckberg	26	0.759 ± 0.404	0.735 (0.480–0.930)
	Cardioplexol™	26	0.923 ± 0.567	0.750 (0.500–1.270)
	Total	52	0.841 ± 0.494	0.745 (0.485–1.026)
≥70	Buckberg	49	1.268 ± 1.089	0.970 (0.590–1.530)
	Cardioplexol™	52	1.065 ± 1.214	0.780 (0.540–1.095)
	Total	101	1.164 ± 1.154	0.820 (0.550–1.230)

**Table 7.2:** Comparison of max. TnT values (ng/ml) in various age groups: PPS population.

Age	Allocation	*n*	mean ± SD	median (IQR)
<65	Buckberg	51	0.791 ± 0.414	0.680 (0.480–1.070)
	Cardioplexol™	34	0.967 ± 0.812	0.750 (0.400–1.140)
	Total	85	0.861 ± 0.607	0.700 (0.480–1.070)
≥65 and <70	Buckberg	26	0.759 ± 0.404	0.735 (0.480–0.930)
	Cardioplexol™	22	0.844 ± 0.517	0.750 (0.500–1.070)
	Total	48	0.816 ± 0.459	0.745 (0.485–0.970)
≥70	Buckberg	49	1.268 ± 1.089	0.970 (0.590–1.530)
	Cardioplexol™	44	0.880 ± 0.460	0.745 (0.535–1.085)
	Total	93	1.085 ± 0.869	0.820 (0.550–1.220)

**Table 7.3:** Comparison of max. TnT values (ng/ml) in various age groups: mod-PPS population.

Age	Allocation	*n*	mean ± SD	median (IQR)
<65	Buckberg	51	0.791 ± 0.414	0.680 (0.480–1.070)
	Cardioplexol™	37	1.022 ± 0.816	0.780 (0.590–1.170)
	Total	88	0.888 ± 0.622	0.710 (0.490–1.085)
≥65 and <70	Buckberg	26	0.759 ± 0.404	0.735 (0.480–0.930)
	Cardioplexol™	25	0.942 ± 0.570	0.760 (0.530–1.270)
	Total	51	0.849 ± 0.496	0.750 (0.490–1.070)
≥70	Buckberg	49	1.268 ± 1.089	0.970 (0.590–1.530)
	Cardioplexol™	49	0.874 ± 0.443	0.780 (0.540–1.080)
	Total	98	1.071 ± 0.851	0.810 (0.550–1.220)

**Table 8.1:** Comparison of max. TnT values (ng/ml) between genders: FAS population.

Gender	Allocation	*n*	mean ± SD	median (IQR)
Male	Buckberg	96	0.877 ± 0.549	0.735 (0.485–1.105)
	Cardioplexol™	83	1.071 ± 1.255	0.740 (0.530–1.140)
	Total	179	0.967 ± 0.946	0.740 (0.500–1.110)
Female	Buckberg	30	1.267 ± 1.241	0.925 (0.590–1.480)
	Cardioplexol™	34	1.261 ± 1.452	0.945 (0.540–1.360)
	Total	64	1.264 ± 1.346	0.935 (0.570–1.435)

**Table 8.2:** Comparison of max. TnT values (ng/ml) between genders: PPS population.

Gender	Allocation	*n*	mean ± SD	median (IQR)
Male	Buckberg	96	0.877 ± 0.549	0.735 (0.485–1.105)
	Cardioplexol™	71	0.838 ± 0.584	0.710 (0.500–1.000)
	Total	167	0.860 ± 0.563	0.710 (0.490–1.070)
Female	Buckberg	30	1.267 ± 1.240	0.925 (0.590–1.480)
	Cardioplexol™	29	1.089 ± 0.639	1.060 (0.610–1.360)
	Total	59	1.179 ± 0.987	0.950 (0.590–1.440)

**Table 8.3:** Comparison of max. TnT values (ng/ml) between genders: mod-PPS population.

Gender	Allocation	*n*	mean ± SD	median (IQR)
Male	Buckberg	96	0.877 ± 0.549	0.735 (0.485–1.105)
	Cardioplexol™	80	0.888 ± 0.609	0.735 (0.515–1.065)
	Total	176	0.882 ± 0.576	0.735 (0.500–1.090)
Female	Buckberg	30	1.267 ± 1.240	0.925 (0.590–1.480)
	Cardioplexol™	31	1.070 ± 0.621	0.950 (0.610–1.360)
	Total	61	1.167 ± 0.973	0.940 (0.600–1.430)

**Table 9.1:** Comparison of max. TnT values (ng/ml) between types of surgery: FAS population.

Type of surgery	Allocation	*N*	mean ± SD	median (IQR)
Isolated CABG	Buckberg	89	0.877 ± 0.524	0.780 (0.520–1.110)
	Cardioplexol™	82	0.909 ± 0.706	0.735 (0.530–1.060)
	Total	171	0.892 ± 0.616	0.740 (0.520–1.090)
Other than isolated CABG	Buckberg	37	1.194 ± 1.178	0.840 (0.480–1.430)
	Cardioplexol™	35	1.636 ± 2.073	1.070 (0.540–1.580)
	Total	72	1.409 ± 1.677	0.935 (0.535–1.520)

**Table 9.2:** Comparison of max. TnT values (ng/ml) between types of surgery: PPS population.

Type of surgery	Allocation	*n*	mean ± SD	median (IQR)
Isolated CABG	Buckberg	89	0.877 ± 0.524	0.780 (0.520–1.110)
	Cardioplexol™	79	0.867 ± 0.584	0.730 (0.500–1.060)
	Total	168	0.872 ± 0.551	0.740 (0.520–1.090)
Other than isolated CABG	Buckberg	37	1.194 ± 1.178	0.840 (0.480–1.430)
	Cardioplexol™	21	1.074 ± 0.683	0.940 (0.530–1.360)
	Total	58	1.150 ± 1.021	0.900 (0.500–1.430)

**Table 9.3:** Comparison of max. TnT values (ng/ml) between types of surgery: mod-PPS population.

Type of surgery	Allocation	*n*	mean ± SD	median (IQR)
Isolated CABG	Buckberg	89	0.877 ± 0.524	0.780 (0.520–1.110)
	Cardioplexol™	81	0.864 ± 0.578	0.730 (0.530–1.060)
	Total	170	0.871 ± 0.549	0.740 (0.520–1.090)
Other than isolated CABG	Buckberg	37	1.194 ± 1.178	0.840 (0.480–1.430)
	Cardioplexol™	30	1.141 ± 0.675	1.005 (0.600–1.440)
	Total	67	1.170 ± 0.979	0.930 (0.530–1.440)

### Secondary efficacy endpoints

CK-MB results were very similar to those observed for TnT (Table 10). Time between aortic cross-clamping and cardiac arrest was significantly shorter after Cardioplexol™ (*p* < 0.001). Although not an endpoint, cross-clamp time was shorter after Cardioplexol™ vs. Buckberg (51.2, 95% CI: 24.2–87.2 min vs. 60.7, 95% CI: 18.8–130.0 min; *p* < 0.001). Other favorable effects of Cardioplexol™: lower rate of defibrillation (10% vs. 52%, *p* < 0.001), reduced post-operative inotropic support (*p* < 0.001), and reduction in ICU stay (38.1 vs. 44.0 h, *p* = 0.110).

**Table 10.1:** Results of secondary endpoints: FAS population.

	Secondary endpoint	Statistics	Cardioplexol™ (*n* = 119)	Buckberg (*n* = 129)	Comparison
1	Maximal value of CK-MB during the first 24 h (U/L)	mean^a^ 95% CI^a^	56.7 (51.0–63.0)	54.0 (48.8–59.8)	1.05^b^ (0.91–1.22)^b^ *p* = 0.510
2	Time between aortic crossclamping and the complete cardiac arrest (sec)	Median range	12 (2–261)	71 (13–596)	*p* < 0.0001^c^
3	Catecholamines during aortic cross-clamping	Yes	118 (99.2%)	128 (99.2%)	*p* > 0.9^d^
4	Cumulative dose of catecholamines during aortic cross-clamping	Median range	779.0 (30–9,270)	785.5 (6–24,877)	*p* = 0.359^c^
5	Defibrillation after aorta unclamping and coronary reperfusion	Yes	15 (12.6%)	66 (51.2%)	*p* < 0.0001^d^
6	Cumulative dose of catecholamines during the first 24 h	Median range	6,000 (178–83,000)	7,395 (329–131,394)	*p* = 0.070^c^
7	Installation of an IABP in the first 24 h	Yes	1	6	*p* = 0.122^d^
8	Duration of intubation (h)	Median range	13.0 (4.5–102)	13.5 (7–480)	*p* = 0.105^c^
9	Duration of ICU stay (h)	Median range	37.8 (7.6–240.1)	43.7 (14.8–503.9)	*p* = 0.284^c^
10	Death during the first 24 h	Yes	1	2	*p* > 0.9^d^
11	Maximal ST elevation during the first 24 h (mm)	Median range	2.0 (0–5)	2.0 (0–6)	*p* = 0.669^c^
12	Duration of hospitalization (days)	Median range	10 (0–19)	11 (2–30)	*p* = 0.139^c^

^a^ Geometric mean and CI based on back transformed (anti-log) CK-MB values.

^b^ Ratio of geometric means and corresponding 95% CI based on back transformed values.

^c^ Ttest for two independent groups.

^d^ Fisher's exact test.

**Table 10.2:** Results of secondary endpoints: PPS population.

	Secondary endpoint		Cardioplexol™ (*n* = 100)	Buckberg (*n* = 126)	Comparison
1	Maximal CK-MB value (first 24 h, U/L)	mean^a^ 95% CI^a^	51.6 (45.5–57.1)	54.2 (49.4–59.4)	0.95^b^ (0.83–1.09)^b^ *p* = 0.483
2	Time between aortic cross-clamping and complete cardiac arrest (s)	Median range	11 (2–261)	71 (13–551)	*p* < 0.0001^c^
3	Catecholamines during aortic cross-clamping	Yes	99 (99.0%)	125 (99.2%)	*p* > 0.9^d^
4	Cumulative dose of catecholamines during aortic cross-clamping	Median range	816 (30–9,270)	791 (6–24,877)	*p* = 0.200^c^
5	Defibrillation after aorta unclamping	Yes	10 (10.0%)	65 (51.6%)	*p* < 0.0001^d^
6	Cumulative dose of catecholamines (first 24 h)	Median range	6,202 (178–70,800)	7,170 (329–131,394)	*p* = 0.070^c^
7	Installation of an IABP in the first 24 h	Yes	1	5	*p* = 0.068^d^
8	Duration of intubation (h)	Median range	13.0 (4.5–102)	13.5 (7–480)	*p* = 0.111^c^
9	Duration of ICU stay (h)	Median range	38.1 (13.1–173.3)	44.0 (14.8–503.9)	*p* = 0.110^c^
10	Death during the first 24 h	Yes	–	1	*p* > 0.9^d^
11	Maximal ST elevation during the first 24 h (mm)	Median range	2.0 (0–5)	2.0 (0–5)	*p* > 0.9^c^
12	Duration of hospitalization (days)	Median range	10 (7–19)	11 (2–30)	*p* = 0.035^c^

^a^ Geometric mean and CI based on back transformed (anti-log) CK-MB values.

^b^ Ratio of geometric means and corresponding 95% CI based on back transformed values.

^c^ Ttest for two independent groups.

^d^ Fisher's exact test.

**Table 10.3:** Results of secondary endpoints: mod-PPS population.

	Secondary endpoint	Statistic s	Cardioplexol™ (*n* = 111)	Buckberg (*n* = 126)	Comparison
1	Maximal value of CK-MB during the first 24 h (U/L)	mean^a^ 95% CI^a^	52.7 (47.8–58.1)	54.2 (49.4–59.4)	0.97 (0.85–1.11) *p* = 0.684
2	Time between aortic crossclamping and the complete cardiac arrest (sec)	Median range	12 (2–261)	71 (13–551)	*p* < 0.0001^c^
3	Catecholamines during aortic cross-clamping	Yes	110 (99%)	125 (99%)	*p* > 0.9^d^
4	Cumulative dose of catecholamines during aortic cross-clamping	Median range	779 (30–9,270)	791 (6–24,877)	*p* = 0.218^c^
5	Defibrillation after aorta unclamping and coronary reperfusion	Yes	13 (12%)	65 (52%)	*p* < 0.0001^d^
6	Cumulative dose of catecholamines during the first 24 h	Median range	6,000 (178–70,800)	7,170 (329–131,394)	*p* = 0.030^c^
7	Installation of an IABP in the first 24 h	Yes	0	5	*p* = 0.062^d^
8	Duration of intubation (h)	Median range	13.0 (4.5–102)	13.5 (7–480)	*p* = 0.094^c^
9	Duration of ICU stay (h)	Median range	37.8 (12.2–173.3)	44.0 (14.8–503.9)	*p* = 0.147^c^
10	Death during the first 24 h	Yes	0	1	*p* > 0.9^d^
11	Maximal ST elevation during the first 24 h (mm)	Median range	2.0 (0–5)	2.0 (0–5)	*p* > 0.9^c^
12	Duration of hospitalization (days)	Median range	10 (7–28)	11 (2–30)	*p* = 0.105^c^

^a^ Geometric mean and CI based on back transformed (anti-log) CK-MB values.

^b^ Ratio of geometric means and corresponding 95% CI based on back transformed values.

^c^ Ttest for two independent groups.

^d^ Fisher's exact test.

### Secondary safety endpoints

Follow-up results at 30 days were comparable between the groups (Table 11) except for mortality (1 Cardioplexol™ vs. 5 Buckberg patients). Adverse events, severity grade and causality are summarized in Supplementary Appendices 4, 5. Number of patients with adverse events was similar in both groups. Number of adverse events was however slightly lower in the Cardioplexol™ group.

**Table 11:** Follow-up results at 30 days post-surgery (safety population).

	Cardioplexol™ (*N* = 119)	Buckberg (*N* = 129)
Patient alive at discharge or 30 days post-surgery (yes)	118 (99%)	124 (96%)
Patient still hospitalized (yes)	0	0
Death	1 (1%)	5 (4%)
IABP/assist device (yes)	1 (1%)	2 (2%)
Dialysis (yes)	0	0
Tamponade necessitating a drainage (yes)	0	3 (2%)
Resternotomy for hemostasis (yes)	0	0
Resternotomy due to hemodynamic instability (yes)	0	0
ECG, new Q wave (yes)	2 (2%)	2 (2%)
Neurologic complication (yes)	4 (3%)	9 (7%)
Arrhythmic complication (yes)	32 (27%)	30 (23%)
Potential adverse event other than any of above (yes)	63 (53%)	67 (52%)

In both groups, pH, lactate, haematocrit, potassium, sodium and calcium values remained in normal ranges during the entire procedure (Table 12). Blood transfusion was deemed necessary in 22% of patients operated on with Cardioplexol™ and 29% of patients operated on with Buckberg.

**Table 12:** Intraoperative blood gas analysis at the specified time after termination of initial cardioplegic infusion/injection (safety population).

	Time point	Cardioplexol™ (*N* = 119)		Buckberg (*N* = 129)	
	(min)	No. assessed/median (min, lqr, uqr, max)			
pH	Pre	118	7.40 (7.29, 7.37, 7.43, 7.55)	128	7.40 (7.28, 7.36, 7.42, 7.51)
	5	118	7.41 (7.30, 7.38, 7.43, 7.53)	129	7.40 (7.29, 7.37, 7.43, 7.55)
	30	119	7.39 (7.25, 7.37, 7.42, 7.53)	127	7.39 (7.29, 7.36, 7.42, 7.49)
	60	97	7.39 (7.28, 7.36, 7.41, 7.50)	98	7.38 (7.27, 7.36, 7.41, 7.50)
	90	25	7.39 (7.30, 7.36, 7.41, 7.48)	34	7.38 (7.29, 7.36, 7.40, 7.45)
	120	0	–	9	7.40 (7.33, 7.36, 7.40, 7.47)
	150	0	–	1	7.38
	180	0	–	1	7.34
	210	0	–	1	7.40
Lactate (mmol/L)	Pre	117	0.90 (0.40, 0.80, 1.20, 3.30)	128	1.00 (0.50, 0.80, 1.30, 2.20)
	5	118	1.20 (0.60, 1.00, 1.80, 4.50)	129	1.20 (0.60, 0.90, 2.10, 3.80)
	30	119	1.30 (0.70, 1.10, 1.80, 3.50)	126	1.40 (0.60, 1.10, 1.90, 3.80)
	60	97	1.50 (0.90, 1.20, 1.90, 6.10)	98	1.70 (0.70, 1.30, 2.30, 4.70)
	90	25	1.80 (0.90, 1.20, 2.30, 4.90)	34	1.80 (0.70, 1.30, 2.40, 3.60)
	120	0	–	9	1.80 (1.00, 1.60, 3.00, 3.40)
	150	0	–	1	5.00
	180	0	–	1	4.90
	210	0	–	1	4.50
Ht (%)	Pre	118	37 (27, 33, 40, 47)	128	37 (23, 34, 39, 47)
	5	117	30 (20, 27, 32, 39)	129	29 (19, 27, 32, 38)
	30	119	30 (22, 27, 32, 38)	127	29 (20, 27, 32, 39)
	60	97	30 (22, 28, 32, 37)	98	29 (21, 26, 32, 37)
	90	25	30 (23, 27, 32, 36)	34	28 (20, 25, 30, 37)
	120	0	–	9	28 (24, 26, 28, 31)
	150	0	–	1	31
	180	0	–	1	25
	210	0	–	1	22
Na+ (mmol/L)	Pre	118	140 (132, 138, 142, 148)	128	140 (135, 139, 141, 145)
	5	118	138 (130, 137, 140, 144)	129	137 (131, 136, 139, 147)
	30	119	139 (132, 137, 140, 144)	127	137 (129, 135, 138, 145)
	60	97	138 (131, 137, 140, 143)	98	137 (128, 135, 138, 146)
	90	25	139 (135, 138, 140, 143)	34	137 (134, 136, 140, 142)
	120	0	–	9	138 (135, 137, 141, 142)
	150	0	–	1	137
	180	0	–	1	138
	210	0	–	1	138
K+ (mmol/L)	Pre	118	4.05 (3.00, 3.80, 4.40, 6.60)	128	4.10 (3.10, 3.75, 4.40, 5.70)
	5	118	4.60 (3.60, 4.30, 4.90, 6.60)	129	4.90 (3.80, 4.60, 5.30, 7.30)
	30	119	4.70 (3.50, 4.30, 5.00, 6.00)	127	4.90 (3.90, 4.50, 5.30, 7.50)
	60	97	4.80 (3.60, 4.40, 5.10, 6.10)	98	4.85 (4.10, 4.40, 5.30, 6.90)
	90	25	4.80 (4.00, 4.30, 5.00, 5.80)	34	4.70 (3.10, 4.40, 5.10, 5.90)
	120	0	–	9	4.30 (4.00, 4.00, 4.60, 5.40)
	150	0	–	1	5.20
	180	0	–	1	4.50
	210	0	–	1	4.40
Ca++ (mmol/L)	Pre	118	1.20 (1.09, 1.17, 1.22, 1.33)	128	1.19 (1.09, 1.16, 1.22, 1.36)
	5	118	1.22 (1.03, 1.18, 1.25, 1.33)	129	1.22 (1.06, 1.18, 1.27, 1.46)
	30	119	1.24 (1.10, 1.21, 1.27, 1.37)	127	1.21 (1.05, 1.17, 1.25, 1.43)
	60	97	1.24 (1.16, 1.20, 1.27, 1.38)	98	1.21 (1.04, 1.17, 1.24, 1.43)
	90	25	1.25 (1.15, 1.21, 1.29, 1.46)	34	1.21 (1.09, 1.17, 1.25, 1.38)
	120	0	–	9	1.20 (1.11, 1.15, 1.23, 1.28)
	150	0	–	1	1.17
	180	0	–	1	1.19
	210	0	–	1	1.15

## Discussion

The current pivotal study aimed at demonstrating the safety and efficacy of Cardioplexol™, a new low volume cardioplegic solution which showed several advantages in previous reports (11, 18). Maximal post-operative TnT values were similar in both the PPS- and mod-PPS-populations, with a non-inferiority margin not exceeding 20% of the Buckberg solution values. In addition, results for all 12 secondary endpoints showed a clear benefit of Cardioplexol™, especially regarding the time between aortic cross-clamping and cardiac arrest, the defibrillation rate following aortic unclamping, the cumulative dose of catecholamines within 24 h, post-operative ICU stay, and length of hospital stay. The study also demonstrated the safety of Cardioplexol™ when used as recommended.

### Non-inferiority analysis

From a strictly regulatory standpoint, the recommended approach for noninferiority trials is to perform both FAS and PPS analyses, and to conclude noninferiority only if both give the same results. Since a relevant number of patients had to be excluded, making the PPS-population possibly different from the original FAS-populations, the regulatory authorities argued that a potential bias could have been introduced with consequences on the overall interpretation. However, conditions in the current study need to be put into perspective. Indeed, it is important to carefully assess the relevance of protocol deviations likely to occur in such a trial. Clearly, a population that excludes non-compliant patients (PPS-population), and that properly addresses the impact of these data, is more likely to provide reliable non-inferiority results. Conversely, a population that better matches daily-life scenario (mod-PPS-population), will better reflect clinical reality to be expected once the drug is commercially available. In a real-life scenario, surgeons decide on an individual basis which strategy is most appropriate. This is particularly true for a study such as this one, where a well-established treatment is compared to a new one with which surgeons have no experience so far. For that reason, it was reasonable to expect only minor deviations in the Buckberg group, without effect on the primary endpoint. Accordingly, Buckberg administration was not strictly monitored but left to the surgeon's discretion. In contrast, Cardioplexol™ administration was strictly formulated, and all surgeons received some theoretical training. Protocol violations were recorded in 19 cases. Some may be considered “mild” (missing postoperative TnT values, *n* = 2; delayed administration of the second/third dose, *n* = 11), with only little influence on primary endpoint. For 6 patients, reasons for exclusion were more serious with potential major impact on primary endpoint: 3 patients also received Buckberg (cross-over), and 3 patients had serious administration errors: volume too low (85 ml), volume too high (>200 ml), injection too slow (197 s). The mod-PPS population excluded only these later 6 patients.

### Primary endpoint

Several studies indicate that postoperative TnT profile correlates well with short-, medium- and even long-term prognosis (16, 19–21). Timing of peak occurrence may reflect different clinical scenarios (22). It typically occurs around 6–8 h post myocardial reperfusion in uncomplicated cardiac surgery procedures (22–30). Conversely, in cases of acute coronary syndrome without coronary reperfusion, the increase in TnT typically extends over 24 h (31). Therefore, post-operative TnT values that keep increasing after 12 or 24 h likely correspond to an event that was not resolved by reperfusion. This might be cardioplegia-related but could also reflect an independent issue (coronary artery occlusion, graft thrombosis, kinking or twist).

Maximum and 6 h reperfusion values seem thus to best reflect quality of cardioplegic protection. Both values are clearly non-inferior as compared to those observed in the Buckberg group.

### Secondary endpoints

Although less specific and sensitive than TnT, CK-MB remains a traditional biomarker used to evaluate cardioplegic solutions (32–34). In the present study, CK-MB values appeared similar in both groups.

Other secondary endpoints were selected according to their direct or indirect relationship with clinical outcome. For instance, a rapid cardiac arrest critically limits the metabolic demands of non-perfused heart (35–38) and improves myocardial integrity during the ischemic period. In the present study, cardiac arrest occurred much faster after Cardioplexol™ and the positive consequences was reflected by a significant reduction of ventricular fibrillation after reperfusion, easier conversion (less electrical energy and fewer shocks required; data not shown) and reduced need for post-operative inotropic support. These benefits ultimately led to a reduction in overall ICU length of stay.

The quantity of vasoactive drugs required during the clamping period may reflect hemodynamic changes possibly induced by the cardioplegic solution (32, 34, 39). In present study, an advantage was observed after Cardioplexol™ and can be explained by the small volume, confined to the coronary system. Mortality was assessed at 24 h to better evaluate a possible effect of cardioplegia. Mortality, however, is more commonly assessed at day 30 (4, 33). In present study, only one (Buckberg) patient died within 24 h of surgery (Table 10). At 30-day mortality markedly increased in Buckberg patients but remained low after Cardioplexol™ (Table 11).

### Safety aspects

Distribution and severity of adverse events were homogeneous in both groups. Mortality was however lower after Cardioplexol™. Although Cardioplexol™ contains high equivalent of potassium, serum values remained within a normal range. In fact, they were slightly lower than values in the Buckberg group, probably because the low volume of Cardioplexol™ doses remain confined to the coronary system.

A major deviation from the administration protocol was observed in 6 patients, raising questions about the safety of Cardioplexol™ administration. In principle, administration of Cardioplexol™ is straightforward. However, it differs from standard solutions in its limited volume, rapid direct injection by the surgeon himself, immediate cardiac arrest and no need to repeat administration every 20 min. This prompted a complementary clinical study, aimed at validating a clearly structured training program for surgeons with no prior experience of using Cardioplexol™ (40).

## Limitations

The present study was monocentric and therefore cannot exclude the possibility of it being non-replicable in other centers. Although most confounding factors were well balanced between study groups, confirmation of findings in other centers would be welcome. Furthermore, surgical indications were varied, and essentially included isolated CABGs, valves procedures or a combination of the two, these accounting for the vast majority of current cardiac surgeries. It is known that the benefit of cardioplegia is not always the same in all cases. In addition, post-operative TnT values are known to slightly differ after CABG vs. valve replacement. However, the aim of this pivotal study was to verify that Cardioplexol™ is generally effective, whatever the indication. An initial sub-analysis confirmed the differences in post-operative TnT max values after isolated CABG vs. any operation other than isolated CABG. However, these results remain similar irrespective of the cardioplegia solution adopted (Table 9). Similarly, there were no notable differences across genders or age groups (Tables 7, 8).

Finally, given that the recruitment period extended to 2015, the presentation of the results of this study appears to be relatively delayed. The data analysis was in fact carried out upon completion of the study and submitted to the registration authorities. The data had also been published on the EU clinical trials register website: https://www.clinicaltrialsregister.eu/ctr-search/trial/2011-004198-10/results. The authorities, however, requested that a new study be conducted before the results of this first study could be validated. Indeed, although it considered that the Cardioplexol™ solution was in itself effective and safe, its administration remained a critical point and an administration error could be harmful. In this context, it was requested that a training protocol for surgeons new to the use of Cardioplexol™ be tested in a new phase 3 study, which would therefore be considered complementary to the present study and would enable a final decision to be made regarding marketing authorization. This study has been carried out (40) and confirms that specific training for surgeons who do not yet have experience with the use of Cardioplexol™ helps to avoid administration errors and consequently increases the safety of this medication.

## Conclusion

Safety and efficacy of Cardioplexol™ were confirmed in this pivotal singlecentre, single-blind, randomized Phase-3, non-Inferiority study. Together with the data presented in the supplementary study (40), the results presented here constituted a key part of the European registration dossier. Cardioplexol™ received marketing authorization in Switzerland in September 2023 and in 10 European countries in April 2024.

## Data Availability

The datasets presented in this study can be found in online repositories. The names of the repository/repositories and accession number(s) can be found in the article/Supplementary Material.
