# Supplementary material for: Single-centre, single-blind, randomized, active-controlled phase-3 non-inferiority study to investigate the safety and efficacy of the cardioplegic solution Cardioplexol™
Source: Front Cardiovasc Med. 2025 May 15;12:1587713. doi: 10.3389/fcvm.2025.1587713 (PMC12128416; doi:10.3389/fcvm.2025.1587713)
Supplement: Supplementary file 1 [file Table1.docx]

**A Single-Centre, Single Blind, Randomized, Active-Controlled Phase 3 Non-Inferiority Study to Investigate the Safety and Efficacy of the Cardioplegic Solution Cardioplexol™**

***Appendix***

***Appendix 1***

Randomized patients who were not operated and therefore not included in the FAS population (n=17).

| **Buckberg (n=4)** | |
| --- | --- |
|  | **Surgeon chose another option** |
| 1 | Surgeon decided to use a different cardioplegic solution (Custodiol). |
|  | **Consent withdrawn** |
| 2 | Patient or legally authorized representative withdrew consent. |
|  | **Eligibility for study inclusion no longer given** |
| 3 | Shortly before surgery radiology informed the surgeon of an aortic aneurysm that was not diagnosed in routine pre surgery check but was visible on the CT scan made the day before surgery. Surgery required then an additional full aortic arch replacement with full circulatory arrest |
|  | **Change in OP schedule** |
| 4 | Due to a change in the OP schedule (OP was moved forward) no study nurse was available |
| **Cardioplexol™ (n=13)** | |
|  | **Surgeon chose another option** |
| 1 | Patient wasn't included because surgeon refused the patient's study participation |
| 2 | Surgeon refused to operate patient in study (LV-hypertrophy) |
| 3 | Surgeon refused to operate patient in study |
| 4 | Surgeon refused to operate patient in study |
| 5 | Surgeon refused to operate patient in study |
| 6 | Surgeon decided to operate off-pump immediately before planned OP |
|  | **Eligibility for study inclusion no longer given** |
| 7 | Intra-operative TEE performed before cannulation showed reduced LVEF of approx. 20-25%, patient no longer eligible |
| 8 | TEE performed at the beginning of surgery, prior to administration of cardioplegia, showed an aortic insufficiency >1 |
| 9 | Patient was randomized, but pre-ECC TEE revealed aortic regurgitation >1. This was not described in preoperative TEE findings |
|  | **Change in OP schedule** |
| 10 | The scheduled surgeon could not operate due to illness. The replacing surgeon does not operate under study protocol with Cardioplexol™ solution |
| 11 | Due to a change in the OP schedule (OP was moved forward) no study nurse was available |
|  | **Temperature Deviation of Cardioplexol™** |
| 12 | Temperature deviation of Cardioplexol™ due to a refrigerator malfunction in the night before the surgery. Cardioplexol™ was not used for the surgery |
| 13 | Prior to surgery a temperature deviation in the refrigerator containing the Cardioplexol solutions was recorded. Therefore, Cardioplexol™ could not be used |

***Appendix 2***

Randomized patients not included in the PPS population (n=22).

|  | **Reason for non-inclusion** | **Observed post-operative max TnT (ng/ml)** |
| --- | --- | --- |
| **Buckberg (n=3)** | |  |
|  | **Missing max. TnT values** |  |
| 1 | No post-operative max. Tn T value | - |
| 2 | No post-operative max. Tn T value | - |
| 3 | No post-operative max. Tn T value | - |
| **Cardioplexol^TM^ (n=19)** | |  |
|  | **Missing max. TnT values** |  |
| 1 | No post-operative max. Tn T value | - |
| 2 | No post-operative max. Tn T value | - |
|  | **Cross-over** |  |
| 3 | Subject also received Buckberg in addition | 0.44 |
| 4 | Subject also received Buckberg in addition | 0.55 |
| 5 | Subject also received Buckberg in addition | 8.72 |
|  | **Incorrect application of the first dose** |  |
| 6 | Incorrect volume of initial cardioplegic infusion (85ml), and expired study medication | 10.00 |
| 7 | Duration of initial Cardioplexol injection > 90 sec (197sec) | 4.58 |
| 8 | Incorrect volume of initial cardioplegic infusion > 200ml | 3.31 |
|  | **Incorrect application of the second/third dose** |  |
| 9 | Incorrect timing of Cardioplexol application (cross-clamp time 60.1 min, no second dose) | 0.80 |
| 10 | Incorrect timing of Cardioplexol application (cross-clamp time 61.4 min, no second dose) | 0.45 |
| 11 | Incorrect timing of Cardioplexol application (cross-clamp time 61.7 min, no second dose) | 0.53 |
| 12 | Incorrect timing of Cardioplexol application (cross-clamp time 62.2 min, no second dose) | 0.91 |
| 13 | Incorrect timing of Cardioplexol application (cross-clamp time 62.4 min, no second dose) | 0.80 |
| 14 | Incorrect timing of Cardioplexol application (cross-clamp time 64.1 min, no second dose) | 1.23 |
| 15 | Incorrect timing of Cardioplexol application (cross-clamp time 68.5 min, no second dose) | 0.82 |
| 16 | Incorrect timing of Cardioplexol application (cross-clamp time 71.0 min, no second dose) | 1.29 |
| 17 | Incorrect timing of Cardioplexol application (cross-clamp time 71.2 min, no second dose) | 2.26 |
| 18 | Incorrect timing of Cardioplexol application (cross-clamp time 91.0 min, no third dose) | 2.29 |
| 19 | Incorrect timing of Cardioplexol application (cross-clamp time 106.3 min, no third dose) | 1.75 |

***Appendix 3***

Details on 11 Cardioplexol^TM^ patients not included in the PPS population due to non-respect of administration timing.

|  | **Type of surgery** | **Initial dose (ml)** | **Cross-clamp time (min)** | **TnT-6 hours (ng/ml)** | **Max TnT (ng/ml)** |
| --- | --- | --- | --- | --- | --- |
| **Second dose was not administered** | | | | | |
| 1 | Aortic valve | 100 | 60.1 | 0.80 | 0.80 |
| 2 | Aortic valve | 100 | 61.4 | 0.45 | 0.45 |
| 3 | CABG | 150 | 61.7 | 0.53 | 0.53 |
| 4 | CABG | 100 | 62.2 | 0.91 | 0.91 |
| 5 | Aortic valve | 100 | 62.4 | 0.80 | 0.80 |
| 6 | Aortic valve | 100 | 64.1 | 1.23 | 1.23 |
| 7 | Aortic valve | 150 | 68.5 | 0.82 | 0.82 |
| 8 | Aortic valve | 150 | 71.0 | 1.29 | 1.29 |
| 9 | Aortic valve | 100 | 71.2 | 2.26 | 2.26 |
| **Third dose was not administered** | | | | | |
| 10 | Aortic valve | 100 ^1^ | 91.0 | 2.29 | 2.29 |
| 11 | Aortic valve | 100 ^2^ | 106.3 | 1.75 | 1.75 |

^1^ A second dose of 100 ml was administered after approximately 48 minutes. No third administered.

^2^ In this patient, both the limits of 60 and 90 minutes for the administration of the second and third dose were not respected. The second dose of 100 ml was administered after 61 minutes. No third dose was administered.

***Appendix 4***

Adverse events coded according to MedDRA preferred terms (Safety population) Results include all events reported at least twice during the study.

|  | **Total** | **Cardioplexol^TM^ (n = 119)** | **Buckberg (n = 129)** |
| --- | --- | --- | --- |
| **no. of patients / no. of events** | | | |
| **Total** | 201/499 | 99/226 | 102/273 |
| **Blood and lymphatic system disorders** | | | |
| Anaemia | 90/91 | 45/45 | 45/46 |
| Leukocytosis | 3/3 | 1/1 | 2/2 |
| Thrombocytopenia | 7/7 | 2/2 | 5/5 |
| **Cardiac disorders** | | | |
| Angina pectoris | 3/3 | 2/2 | 1/1 |
| Arrhythmia | 43/44 | 19/19 | 24/25 |
| Atrial fibrillation | 35/36 | 14/14 | 21/22 |
| Atrial tachycardia | 3/3 | 1/1 | 2/2 |
| Atrioventricular block complete | 4/4 | 2/2 | 2/2 |
| Bradycardia | 7/8 | 3/4 | 4/4 |
| Cardiac tamponade | 2/3 | 0/0 | 2/3 |
| Myocardial infarction | 7/7 | 5/5 | 2/2 |
| Pericardial effusion | 3/3 | 0/0 | 3/3 |
| **Endocrine disorders** | | | |
| Hypothyroidism | 2/2 | 2/2 | 0/0 |
| **Gastrointestinal disorders** | | | |
| Constipation | 2/2 | 0/0 | 2/2 |
| Diarrhoea | 6/6 | 2/2 | 4/4 |
| Nausea | 5/5 | 3/3 | 2/2 |
| Vomiting | 5/5 | 1/1 | 4/4 |
| **General disorders and administration site conditions** | | | |
| Death ^1^ | 5/5 | 1/1 | 4/4 |
| Drug ineffective | 5/5 | 3/3 | 2/2 |
| Impaired healing | 7/7 | 4/4 | 3/3 |
| Pain | 4/4 | 2/2 | 2/2 |
| Pyrexia | 2/2 | 1/1 | 1/1 |
| **Immune system disorders** | | | |
| Hypersensitivity | 2/2 | 1/1 | 1/1 |
| **Infections and infestations** |  |  |  |
| Infection | 9/9 | 4/4 | 5/5 |
| **Injury, poisoning and procedural complications** | | | |
| Anaemia postoperative | 19/19 | 12/12 | 7/7 |
| Fall | 2/2 | 2/2 | 0/0 |
| Postoperative thoracic procedure complication | 2/2 | 1/1 | 1/1 |
| Wound secretion | 2/2 | 2/2 | 0/0 |
| **Investigations** | | | |
| Electrocardiogram Q waves | 2/2 | 1/1 | 1/1 |
| **Metabolism and nutrition disorders** | | | |
| Fluid retention | 2/2 | 0/0 | 2/2 |
| Hypoalbuminaemia | 2/2 | 1/1 | 1/1 |
| **Nervous system disorders** | | | |
| Neurologic symptoms | 2/2 | 1/1 | 1/1 |
| Syncope | 2/2 | 1/1 | 1/1 |
| **Psychiatric disorders** | | | |
| Delirium | 6/6 | 2/2 | 4/4 |
| Reactive psychosis | 10/10 | 3/3 | 7/7 |
| **Renal and urinary disorders** | | | |
| Renal failure | 3/3 | 2/2 | 1/1 |
| **Respiratory, thoracic and mediastinal disorders** | | | |
| Atelectasis | 2/2 | 0/0 | 2/2 |
| Bronchospasm | 2/2 | 0/0 | 2/2 |
| Cough | 9/9 | 3/3 | 6/6 |
| Dyspnoae | 3/3 | 2/2 | 1/1 |
| Lung infiltration | 4/4 | 2/2 | 2/2 |
| Pleural effusion | 21/24 | 7/7 | 14/17 |
| Pneumothorax | 8/8 | 5/5 | 3/3 |
| Respiratory failure | 4/4 | 2/2 | 2/2 |
| **Skin and subcutaneous tissue disorders** | | | |
| Dermatitis | 4/4 | 3/3 | 1/1 |
| Dermatitis contact | 2/2 | 2/2 | 0/0 |
| Rash | 2/2 | 1/1 | 1/1 |
| **Vascular disorders** | | | |
| Haematoma | 2/2 | 0/0 | 2/2 |
| Haemodynamic instability | 3/3 | 0/0 | 3/3 |
| Hypertension | 3/3 | 1/1 | 2/2 |
| Hypotension | 3/3 | 2/2 | 1/1 |

^1^ In one case death was reported as an outcome of an AE. In total 6 patients died during the study.

***Appendix 5***

Distribution of adverse events according to their severity grade and causality (Safety population).

|  | **Total** | **Cardioplexol^TM^ ( n = 119)** | **Buckberg (n = 129)** |
| --- | --- | --- | --- |
| **no. of patients/ no. of events** | | | |
| AE | 201/495 | 99/222 | 102/273 |
| SAE | 114/180 | 55/75 | 59/105 |
| **AE Grade** | | | |
| mild | 179/318 | 92/158 | 87/160 |
| moderate | 85/114 | 36/44 | 49/70 |
| severe | 32/45 | 13/18 | 19/27 |
| life-threatening | 8/10 | 1/1 | 7/9 |
| death | 6/8 ^1^ | 1/1 | 5/7 |
| **Causality/relationship according to drug safety office** | | | |
| certain | 1/1 | 0/0 | 1/1 |
| possible | 7/11 | 5/6 | 2/5 |
| unlikely | 19/33 | 6/11 | 13/22 |
| not related | 191/447 | 94/202 | 97/245 |
| not assessable | 2/2 | 2/2 | 0/0 |
| unknown | 1/1 | 1/1 | 0/0 |
| **Causality/relationship according to the investigator** | | | |
| possible | 6/8 | 3/3 | 3/5 |
| unlikely | 17/31 | 5/10 | 12/21 |
| not related | 190/447 | 94/204 | 96/243 |
| unknown | 8/9 | 4/5 | 4/4 |
| Event unexpected | 181/354 | 88/165 | 93/189 |

AE: Adverse event; SAE: Serious adverse event

^1^ Six patients died during the course of the study. However, eight events were listed with a severity grade of “death”.
